# IOL Power Calculation After Laser-Based Refractive Surgery: Measured vs. Predicted Posterior Corneal Astigmatism Using the Barrett True-K Formula

**DOI:** 10.3390/jcm14114010

**Published:** 2025-06-05

**Authors:** Giacomo De Rosa, Daniele Criscuolo, Laura Longo, Davide Allegrini, Mario R. Romano

**Affiliations:** 1 Department of Biomedical Sciences, Humanitas University, Via Rita Levi Montalcini 4, Pieve Emanuele, 20072 Milan, Italy; 2 Eye Center, Humanitas Gavazzeni-Castelli, 24128 Bergamo, Italy; 3 IRCCS Humanitas Research Hospital, 20089 Milan, Italy

**Keywords:** refractive surgery, cataract surgery, intraocular lens, laser vision correction, myopia, hypermetropia, astigmatism, Barrett

## Abstract

**Background/Objectives:** This study assessed the reliability of the Barrett True-K formula in patients who had undergone laser-based corneal refractive surgery by comparing outcomes using measured vs. predicted posterior corneal astigmatism (PCA) within the Barrett True-K No History formula. **Methods**: We selected 49 eyes out of 41 patients with a history of uncomplicated laser visual correction (LVC) that underwent cataract surgery between 2020 and 2024. The Front K1 and K2, the Back K1 and K2, the anterior chamber depth, the lens thickness, the horizontal white-to-white, and the central corneal thickness were measured using Pentacam. The axial length was measured using the IOL Master 500 or NIDEK AL-Scan. These data were then imported into the freely available online Barrett True-K calculator for post-LVC eyes, and the postoperative results were compared with the predicted IOL target. The cumulative distribution of the refractive prediction error, absolute refractive prediction error, and refractive prediction error were calculated as the difference between the postoperative spherical equivalent and the expected spherical equivalent for both the predicted and measured PCA calculations. **Results:** The results suggest improved accuracy with the Barrett True-K formula when incorporating measured PCA values, supporting the use of corneal tomography for optimized refractive outcomes in post-LVC cataract patients. **Conclusions:** It is always advisable to measure the posterior corneal surface using corneal tomography in all patients who have undergone LVC to achieve better refractive outcomes after cataract surgery.

## 1. Introduction

Laser-based refractive surgery for myopia, hyperopia, and astigmatism relies on excimer laser ablation to precisely reshape the anterior surface of the cornea. This process involves altering the corneal anterior radius of the curvature in a controlled manner while leaving the posterior surface of the cornea and its radius untouched [1].

**Posterior corneal astigmatism (PCA)**: It is important to emphasize that total corneal astigmatism is the sum of anterior and posterior corneal astigmatism. The posterior corneal surface, similar to the anterior surface, is toric and not spherical surface, meaning it is characterized by a steeper meridian and a flatter one. In approximately 80% of eyes, the posterior vertical meridian is steeper than the horizontal. However, the posterior cornea acts as a divergent negative lens, creating a net positive refractive power horizontally, leading to against-the-rule (ATR) posterior astigmatism. This small amount of posterior corneal astigmatism has been shown to reduce total corneal astigmatism by about 13% on average [2]. Some studies have suggested that in about 28% of eyes, total corneal astigmatism differs from anterior corneal astigmatism by more than 0.5 diopters (D) or more than 10 degrees in the meridian, and this is due to the contribution of PCA [3].

In modern intraocular lens (IOL) calculations assisted by corneal tomography, it is important to account for the contribution of PCA as this can significantly influence the choice of the final IOL power.

In this scenario, traditional IOL formulas suffer from several significant limitations that may affect their reliability.

Traditional formulas use the anterior keratometry readings to estimate the posterior corneal negative power and determine the total corneal refractive power by using a predetermined refractive index of 1.3375, which relies on the assumption that the posterior–anterior corneal radius ratio is fixed and uniform [4]. This assumption proves to be valuable in virgin non-laser-corrected eyes, where the posterior–anterior ratio is between 82 and 84 percent. However, using this fixed ratio is not reliable in post-LVC eyes because of the laser-induced flattening, or steepening of the anterior surface, altering the effective corneal refractive index approximation used in traditional IOL calculation [5]. This would result in an over-estimation of the corneal power in eyes that have undergone myopic-LVC, leading to unexpected hyperopia after cataract surgery. Conversely, in eyes that have undergone hyperopic LVC, there would be an under-estimation of the corneal power with subsequent postoperative unexpected myopia [6].

Modern biometers collect the keratometry readings by extrapolation from what they measure at a paracentral zone of 3.2 or 2.5 mm from the corneal center, considering a predictable and uniform curvature gradient of the central zone of the cornea. Once again, this is not the case in patients who have undergone LVC because the excimer ablation primarily flattens (in myopic LVC) or steepens (in hyperopic LVC) the anterior surface of the central zone of the cornea, thereby altering the curvature gradient within this 3 mm zone and consequently making the prediction of the K readings unreliable. This leads to an over-estimation of the corneal central zone power in post-myopic eyes and an under-estimation of it in post-hyperopic eyes, contributing to those postoperative refractive surprises discussed above [7].

Another significant source of error in IOL calculation after LVC is the prediction of the effective lens position (ELP). The ELP refers to the **position of the intraocular lens (IOL)** within the eye after cataract surgery, defined as the distance between the lens and corneal surface, influencing the effective refractive power of the eye. ELP prediction, depending on the specific formula, takes into account several of the eye’s anatomic factors including the axial length (AL), the anterior chamber depth (ACD), and the average dioptric power of the central cornea. Concerning the average central K readings, the prediction of the ELP in post-LVC eyes is less reliable and precise as consequence of the flattening or steepening of the corneal central zone after excimer ablation while the AL and ACD remain the same [8].

The combination of these factors makes the IOL calculation in LVC patients using traditional formulas less accurate and repeatable due to the variability of the excimer ablation pattern, the type of ametropia corrected, and the total spherical equivalent corrected. As a result, the actual postoperative refraction can significantly deviate from the predicted preoperative refractive target.

To overcome this problem and enhance the IOL selection accuracy, different formulas specifically designed for post-LVC eyes have been proposed in recent years; among these is the Barrett True-K formula [9].

The key benefit of this formula is the option it provides to manually enter the tomographic values of the posterior corneal surface during the IOL calculation, taking into account the precise refractive influence of the posterior cornea on the total corneal power, even in LVC patients where it is often neglected. This formula allows the user the choice between using the inbuilt PCA (posterior corneal astigmatism) prediction option, which does not require the Back K1 and K2 values to be measured, and manually entering the measured PCA values during the input of biometric parameters, as mentioned above.

In the literature, there is often a lack of clarity on whether predicted or measured PCA values have been used to calculate the predicted spherical equivalents to evaluate the performance of this formula. To address this, we decided to independently evaluate the performance of both options within the Barret True-K formula to better determine which one offers greater reliability and predictive accuracy.

The objective of the study was to assess the refractive accuracy of the Barret True-K formula in the process of IOLs selection for post-LVC patients who underwent cataract surgery and determine that using the measured PCA option, through the manual insertion of the posterior corneal power, ensures a better accuracy and predictability of refractive outcomes compared to using the inbuilt predicted PCA option.

## 2. Materials and Methods

We selected a group of 41 patients and 49 eyes who had undergone uncomplicated laser refractive surgery for myopia, hyperopia, and astigmatism in the previous 20 years including PRK, Lasik, and Femto-Lasik. These patients then underwent uncomplicated cataract surgery at our center between January 2020 and June 2024. Despite the limited number of patients, the level of statistical significance for the primary outcomes was achieved; however, limitations inherent to this number included the reduced ability to detect smaller effect sizes and potential variability in patient characteristics, such as the influence of axial length, the K readings, and other anatomical features of the eye, which may limit the applicability of the findings. We excluded patients affected by corneal, retinal, or optic nerve pathologies; those who developed post-cataract-surgery complications such as cystoid macular edema; and those who did not achieve a postoperative minimum of 10/20 of BCVA. All patients were evaluated using Pentacam (Oculus DE, Wetzlar, Germany) corneal tomography to collect the preoperative Front K1 and K2, the Back K1 and K2, the anterior chamber depth, the lens thickness, the horizontal white-to-white, and the central corneal thickness. The axial length (AL) and other biometric data were collected using IOL Master 500 (Carl Zeiss Meditec AG, Jena, Germany) or NIDEK AL-Scan (Nidek Medical, Gamagori, Japan) through the optical non-contact measurement. These data were then imported into the freely available online Barrett True-K calculator for post-LVC eyes. For each patient, we calculated the IOL power prediction using both the measured PCA algorithm and the predicted PCA algorithm, using the same personal data of each patient. The final subjective refraction after cataract surgery was measured at 1 month after surgery and the actual postoperative refractive results were then analyzed and compared to the two different expected refractions to determine which of the two algorithms more closely matched the actual postoperative refractive outcome.

The surgeries were carried out using a temporal clear corneal main port of 2.2 mm and a 1.0 mm paracentesis. The device used for phacoemulsification was the Bausch&Lomb Stellaris Elite (Bausch&Lomb, New York, NY, USA), and the IOLs implanted were the Bausch&Lomb Adapt AO, the Alcon SA60WF, and the Johnson&Johnson GCB00, depending on the specific case and surgeon’s preferences.

Table 1 is a summary of demographics and characteristics of the included patients.

A total of 49 eyes of 41 different patients, 22 women and 19 men, were included in the study. The keratorefractive procedures used were either LASIK (laser in situ keratomileuisis) or PRK (Photo Refractive Keratectomy). The sample did not include any corneal lenticule extraction procedure. The form of ametropia corrected was myopia in 40 cases and hyperopia in 9 cases, with or without astigmatism.

The average age at time of cataract surgery was 57.2 ± 2.5 years.

We did not include the pre-refractive history for any of the patients to guarantee a more uniform calculation because the history was not available for all of them. We also intentionally chose not to include the pre-LVC refractive history, even for patients for whom it was available, in order to isolate and study the sole influence of the manually entered PCA parameters on the final IOL calculation, regardless of the total SE diopters inputted into the formula. We believe that demonstrating the reliability of the formula even in the absence of refractive history could be of great value in clinical practice, where such information is often unavailable or only partially accessible and lacks reliable documentation.

For each case, we calculated the expected spherical equivalent (SE) in diopters using the measured PCA algorithm and the expected SE using the predicted PCA algorithm.

The SE is a simplified measure used to express the overall refractive power of an eye. It combines the sphere and cylinder components of a refractive error into a single value, making it easier to assess the eye’s refractive power.

The expected spherical equivalent (SE) calculated using both the predicted PCA and the measured PCA algorithms, for each case, was subtracted from the actual SE measured at 1 month postoperatively. These values were then used to determine the mean refractive error (MRE), the mean absolute refractive error (MARE), and the cumulative frequency of the refractive target error. The cumulative frequency of the refractive target error refers to a statistical measure used to analyze the distribution of refractive outcomes after an eye surgery, representing how many patients or eyes achieve a particular level of refractive error (e.g., myopia, hyperopia, or astigmatism) relative to a desired target refraction.

The statistical analysis was conducted using Python (Python Software Foundation, version 3.9.14, September 2022), and a *p*-value less than 0.05 was considered statistically significant.

Considering the paired data (prediction errors for the same eyes using two different methods), to assess if the difference in performance between the two methods was statistically significant, we conducted a paired *t*-test on the absolute prediction errors.

We decided to use the paired *t*-test as it is a parametric test used to compare the means of two related groups or conditions and it is often used to determine if there is a statistically significant difference between the mean values of two related samples

The paired *t*-test was conducted using the absolute prediction errors calculated for each eye using both predicted PCA and measured PCA. These columns represent the absolute differences between the actual postoperative spherical equivalent and the predicted values based on each PCA calculation method. The *p*-value obtained from the paired *t*-test was <0.05.

Considering the small sample of patients, the Wilcoxon signed-rank test was performed as it does not rely on distributional assumptions that might not hold with limited data, confirming the *p*-value < 0.05 and the statistical significance of the findings.

## 3. Results

To better visualize the overall performance of the Barret True-K formula, with or without the manual input of posterior corneal astigmatism, we first decided to build a table representing the cumulative frequency of the refractive prediction error. We categorized patients based on the refractive prediction error, calculated as the difference between the actual postoperative SE and the expected preoperative SE. The smaller the difference was, the greater the accuracy was.

Table 2 is a summary of the cumulative frequency of refractive target error expressed in diopters.

We outlined a statistically significant difference (*p* < 0.05) detected at thresholds of ±0.25 D and ±0.50 D, where the measured PCA option demonstrated a higher percentage of patients with a refractive error included in the two intervals.

**Figure 1:**
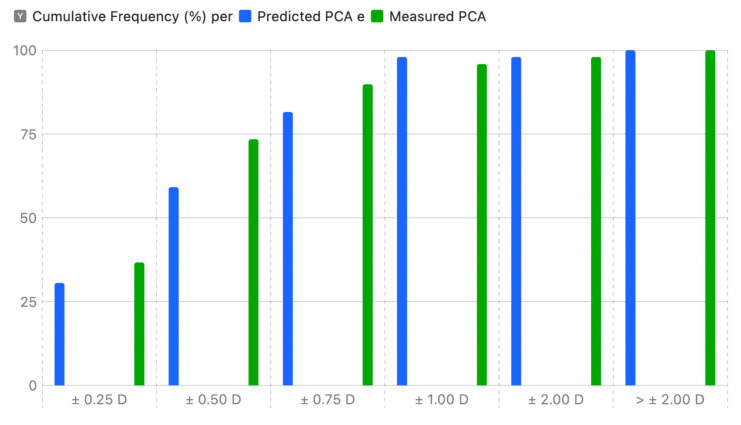
Stacked bar chart representing the cumulative frequency of the refractive error calculated as actual postoperative SE—expected preoperative SE for each method.

To present this distribution more clearly, we provide a stacked bar chart that compares the cumulative frequencies of the predicted PCA and measured PCA calculations (Figure 1).

The stacked bar chart provides a clear and immediate graphical representation of the frequency distribution of individual observations. Analyzing the results, the green bars representing the measured PCA reached 36.7% cumulative frequency in the ±0.25 D category and 73.4% in the ±0.50 D category. In contrast, the blue bars representing the predicted PCA were limited to 30.6% cumulative frequency in the ±0.25 D category and 59.1% in the ±0.50 D category. This suggests that the measured PCA option showed a higher accuracy in achieving postoperative outcomes within tighter refractive error margins.

To assess the accuracy of the BTK with or without the manual PCA values, the values of the mean refractive error, the mean absolute refractive error, and the median are displayed in Table 3, dividing them in two categories based on the calculation option used.

The predicted PCA option showed a mean refractive error of −0.45 diopters with a standard deviation of 0.61 while the measured PCA MRE was −0.28 diopters, with a standard deviation of 0.55.

These findings outline the prominence of the measured PCA option, which significantly reduced the MRE compared to the predicted PCA option (*p* < 0.05).

**Figure 2:**
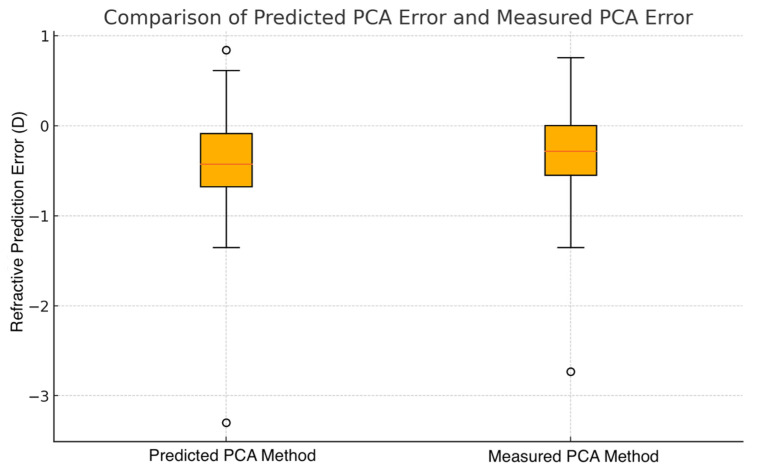
Box plot representing the distribution of refractive prediction error for each method.

To better visualize the distribution of the refractive prediction errors, we present Figure 2.

The median absolute prediction error, represented by the horizontal red line within each box, was notably lower for the measured PCA method compared to the predicted PCA method. The interquartile range (IQR), represented by the height of the box, was also smaller for the measured PCA.

**Scheme 1:**
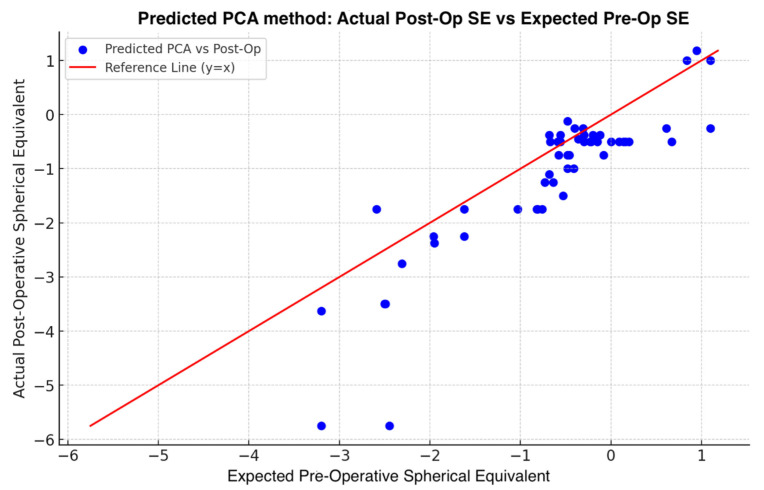
This scheme represents the set of individual observations and the degree of agreement between the preoperative expected SE calculated using the predicted PCA method and the actual postoperative SE, collected 1 month after the surgery.

**Scheme 2:**
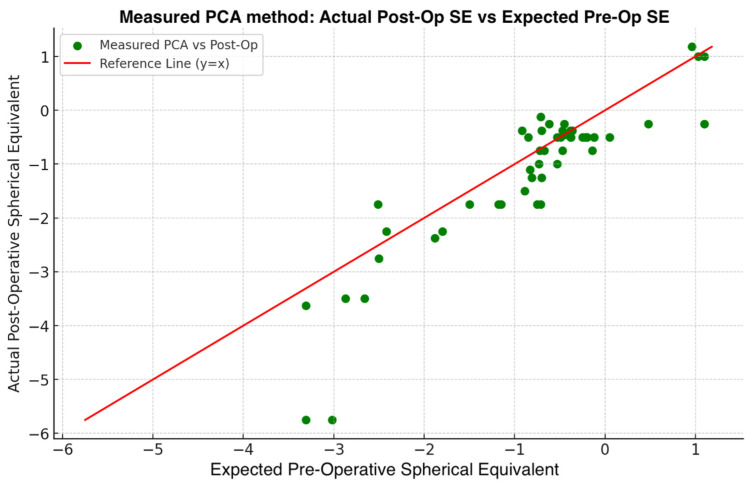
This scheme represents the set of individual observations and the degree of agreement between the preoperative expected SE calculated using the measured PCA method and the actual postoperative SE, collected 1 month after the surgery.

The scatter plot allows for a direct visual comparison of how closely each method’s preoperative prediction aligned with the actual postoperative outcomes. The 45-degree red line represents perfect agreement. In Scheme 1, the green points representing the predicted PCA are more dispersed and farther from the red 45-degree reference line, indicating a greater postoperative refractive error. In contrast, in Scheme 2, the green points cluster more closely around the red 45-degree line, indicating a better agreement with the actual postoperative SEs. The differences observed between the two scatter plots in the distribution of individual data points are not immediately apparent. This is likely due to the fact that, as discussed later, both methods demonstrated satisfactory accuracy, thereby ensuring a small margin of error between the preoperative calculated refractive target and the actual postoperative refractive outcome achieved.

## 4. Conclusions

The valuable performance of the Barret True-K formula is greatly documented in the literature, proving this formula to be reliable and precise in both myopic and hyperopic LVC patients, as demonstrated by Zollet et al. [9,10,11]. Recent studies have highlighted the remarkable performance of the Barrett True-K formula, both with and without history, demonstrating, on several occasions, a lower mean refractive prediction error and a lower mean absolute error compared to other post-LVC formulas [12].

However, in the literature available so far, there has often been a lack of characterization regarding the accuracy of the inbuilt BTK’s posterior corneal astigmatism prediction algorithm, which was the interest of our study. Across the studies available in the literature, there was often no clarity on whether the predicted or the measured PCA options had been used to determine the predicted spherical equivalents. Consequently, we decided to separately test the performances of these two distinct options within the same formula to better understand which one tended to demonstrate greater reliability and prediction accuracy and whether the inbuilt option for the prediction of the posterior corneal astigmatism could guarantee accurate results.

Being aware that the patient sample was limited, and considering the potential variability due to the differences in patient characteristics such as the axial length, keratometric readings, and other anatomical features of the eye, the findings of this study suggest that the Barret True-K method with the manually measured PCA option is more accurate and reliable in determining the IOL power in patients after LVC, thereby limiting the difference between the predicted spherical equivalent and the actual postoperative refraction. We therefore consider the limited number of patients analyzed as the main potential source of reduced reliability in the conclusions drawn from our study. A small sample size inevitably restricted the variability in anatomical and surgical characteristics among individual patients, particularly in terms of the range of refractive errors and, consequently, the total SE diopters ablated by the laser, as well as different corneal morphologies and the possible combinations of anterior and posterior corneal astigmatism in each patient. Although it showed a slightly higher postoperative refractive error, the predicted PCA option still demonstrated a high accuracy, with error rates comparable to those observed in other studies, confirming findings already established in the literature [13].

However, we must consider some other limitations, including that the study relied on Pentacam scheimpflug tomography and IOL Master 500 or NIDEK AL-Scan for biometry data collection. While these devices are widely used, small variations in corneal tomography or axial length measurements could affect the accuracy of IOL power calculation, particularly in post-LVC patients [14]. It would be of great help to plan future studies using newer measurement instruments for the axial length (AXL), anterior keratometry K1 and K2, and posterior keratometry values, thus including the measurement of posterior corneal astigmatism (PCA), to determine the extent to which the postoperative refractive error observed in this study could be attributed to intrinsic limitations of the BTK IOL calculation formula, as opposed to the error arising from the measurement inaccuracy of the instruments employed during the preoperative assessment. To this end, we hope that the researchers will be able to carry out a similar study using anterior segment OCT devices such as the MS39 (CSO, Ferrara, Italy), or Wavefront Aberrometry, to refine the PCA measurement.

Lastly, the study did not account for pre-refractive surgery history, which could have helped in refining the formula’s predictions, especially in cases with significant variations in the corneal curvature due to different amounts of ametropia diopters corrected.

What this study aimed to highlight was the importance of measuring the posterior corneal astigmatism in patients who had undergone LVC prior to cataract surgery; therefore, all surgeons should, to ensure better postoperative refractive outcomes, follow these guidelines:

- Measure posterior corneal astigmatism in all post-LVC patients.
- Use corneal tomography for better IOL selection accuracy.
- Understand the limitations of traditional IOL formulas in post-LVC patients.
- Avoid making the mistake of using traditional formulas for post-LVC patients as this could lead to refractive surprises.
- Always identify the type of refractive surgery performed (whether myopic or hyperopic) using tomographic maps, even if the clinical history is not available.

Although the predicted PCA option offers satisfactory accuracy, the Barret True-K formula using the measured PCA option demonstrates higher accuracy by showing a smaller discrepancy between the actual postoperative SE and the predicted preoperative SE. For surgeons, these findings have significant clinical relevance when managing high-demanding expectations for patients who have undergone LVC. By using advanced formulas like the Barrett True-K formula with measured PCA, surgeons can reduce the likelihood of these errors and better satisfy patient expectations.

Therefore, the final message is that it is always advisable to measure the posterior corneal surface using corneal tomography in all patients who have undergone LVC. This practice allows for the manual entry of posterior corneal astigmatism values into the formula, enhancing overall accuracy and achieving better postoperative refractive outcomes with greater predictability.

## Figures and Tables

**Table 1:** Patient demographics and characteristics at baseline.

Parameter	Mean ± SD	Min	Max
Age (y)	57.2 ± 2.5	45	75
AXL (mm)	25.98 ± 2.35	21.1	34.07
K1 Front (D)	40.41 ± 2.93	32.7	46
K2 Front (D)	40.64 ± 5.85	34.8	46.9
K1 Back (D)	−5.51 ± 2.43	−5.1	−6.4
K2 Back (D)	−6.07 ± 1.84	−5.5	−6.9
ACD (mm)	3.53 ± 0.52	2.49	4.91
IOL Power (D)	19.18 ± 2.96	10.5	25.5
Postoperative SE (D)	−1.104 ± 1.17	−5.75	1.00

AXL = axial length, K1 = flat-axis keratometric value, K2 = steep-axis keratometric value, ACD = anterior chamber depth, IOL = intraocular lens, and SE = spherical equivalent.

**Table 2:** Cumulative frequency of the refractive target error for the two algorithms.

Formula	±0.25 D	±0.50 D	±0.75 D	±1.00 D	±2.00 D	>±2.00 D
Barrett True-K Predicted PCA	15 (30.6%)	29 (59.1%)	40 (81.6%)	48 (97.8%)	48 (97.8%)	49 (100%)
Barrett True-K Measured PCA	18 (36.7%)	36 (73.4%)	44 (89.7%)	47 (95.9%)	48 (97.8%)	49 (100%)

**Table 3:** Mean refractive error (MRE) and mean absolute refractive error (MARE).

Formula	MRE ± SD	MARE ± SD	Median	Min	Max
Barrett True-K Predicted PCA	−0.45 ± 0.61	0.53	−0.425	−3.3	0.84
Barrett True-K Measured PCA	−0.28 ± 0.55	0.43	−0.28	−2.73	0.76

Table 3 is a summary of the mean refractive error (MRE) and the mean absolute refractive error (MARE) for both options, calculated as the difference between the actual postoperative SE and the expected preoperative SE: MRE = Actual SE − Expected SE. MARE = Absolute Actual SE − Absolute Expected SE.

## Data Availability

The original contributions presented in this study are included in the article. Further inquiries can be directed to the corresponding author.
